# Joubert syndrome: Molecular basis and treatment

**DOI:** 10.34763/jmotherandchild.20222601.d-22-00034

**Published:** 2023-02-22

**Authors:** Lidvana Spahiu, Emir Behluli, Violeta Grajçevci-Uka, Thomas Liehr, Gazmend Temaj

**Affiliations:** Department of Pediatrics, University of Prishtina, Prishtina, Kosovo; Institut für Humangenetik, Universitätsklinikum Jena, Friedrich Schiller Universität, Jena, Germany; Human Genetics, College UBT, Faculty of Pharmacy Prishtina, Prishtina Kosovo

**Keywords:** Joubert syndrome (JS), clinical and molecular diagnosis, ciliopathies, therapeutic options

## Abstract

Joubert syndrome (JS; MIM PS213300) is a rare genetic autosomal recessive disease characterized by cerebellar vermis hypoplasia, a distinctive malformation of the cerebellum and the so-called “molar tooth sign.” Other characteristic features are hypotonia with lateral ataxia, intellectual disability/mental retardation, oculomotor apraxia, retinal dystrophy, abnormalities in the respiratory system, renal cysts, hepatic fibrosis, and skeletal changes. Such pleiotropic characteristics are typical of many disorders involving primary cilium aberrations, providing a significant overlap between JS and other ciliopathies such as nephronophthisis, Meckel syndrome, and Bardet-Biedl syndrome. This review will describe some characteristics of JS associated with changes in 35 genes, and will also address subtypes of JS, clinical diagnosis, and the future of therapeutic developments.

## Introduction

Joubert syndrome (JS, **MIM PS213300**) is a neurodevelopment disease which is characterized by malformation in the cerebellum and brainstem, recognizable on axial brain magnetic resonance imaging (MRI) as a “molar tooth sign” [1, 2]. This appearance is the result of an abnormal combination of cerebellar vermis aplasia/hypoplasia, thick and horizontal oriented superior cerebellar peduncles, and sometimes a deep interpeduncular fossa. JS patients typically present as infants with hypotonia, abnormal eye movement, respiratory problems, and ataxia [3, 4, 5]. JS is a multisystem disorder and is manifested in other body systems such as the eyes, kidney, liver, and skeleton [6]. JS is often initially diagnosed in the setting of a neurology, genetics, or pediatric clinic. The purpose of this review is to describe the molecular basis (clinical diagnosis) of JS and to summarize therapeutic approaches.

JS is a very rare genetic disease with a prevalence of 1:55.000-1:200.000 [7, 8]. It is inherited predominantly in an autosomal recessive way and was first described in small, isolated groups with enhanced consanguinity, such as French-Canadian, Ashkenazi Jewish, and Arab populations [9, 10, 11, 12]. JS was first described by Joubert et al. in 1969, who observed it in a French-Canadian family with distant consanguinity related to a common founder 10 generations prior, originating from France (1). JS is part of the large group of diseases called ciliopathies, based on genetic features and overlapping phenotypes related to dysfunction of the primary cilium [13].

### Clinical subtypes of Joubert syndrome

Based on some characteristics that we encounter in patients with this syndrome, JS is classified into several subtypes, as:

a) Classic JS

Classic JS has three main diagnostic findings: oculomotor apraxia, respiratory abnormalities, and polydactyly [8].

b) JS with retinal diseases

This subtype of JS is associated with retinopathy, leading to blindness [14] and/or retinitis pigmentosa with retinal degeneration [15]. It is found in 24-32% of individuals with JS. Retinal dystrophy is linked with genes *AHI1*, *ARL3*, and *CEP290* [14, 16, 17, 18].

c) JS with renal diseases

This subtype can manifest in two forms: NPHP (nephronophthisis) and cystic kidney with corticomedullary cysts, kidney atrophy and interstitial, small, scarred kidneys as pathological features [20,21]. NPHP is a chronic tubule-interstitial nephropathy [19].

d) JS with occulo-renal diseases (Senior-Loken syndrome)

This combination of the previous two subtypes is associated with several gene mutations at a time being involved in JS [22, 23].

e) JS with hepatic diseases (JS 7/ Meckel syndrome 5/ COACH syndrome)

JS with congenital hepatic fibrosis with ductal malformation and dysfunction of cilia has several designations (JS 7/ Meckel syndrome 5/COACH syndrome) and is caused by mutations in the *RPGRIP1L* gene [24]. This sub-type is associated with portal hypertension, elevated liver enzymes, recurrent cholangitis, and gastroesophageal variceal bleeding with thrombocytopenia.

f) JS with oral-facial-digital features

Here, JS is associated with a cleft lip and palate, midline groove of the tongue, gum (tongue) hamartomas, hypertelorism, and micrognathia. Polydactyly is often postaxial, although preaxial polydactyly is not uncommon, and is found in combination with polydactyly on hands and feet [23].

g) JS with acrocallosal features

This sub-type is associated with abnormal corpus callosum connecting two cerebral hemispheres. On an MRI scan, up to 80% of patients with JS had some sort of callosal dysgenesis [25].

h) JS with Juene asphyxiating dystrophy

For this sub-type, characteristics are polydactyly, narrow thoracic rib cage, short ribs, shortened tubular bone, and “trident” appearance in acetabular roof with or without polydactyly. Other features are short stature, rhizomelic limb shortening, cone shape phalangeal epiphyses, and branchydactyly [26, 27, 28].

### Clinical diagnosis of JS

There are three criteria for diagnosis of JS: 1) the “molar tooth sign” (MTS); 2) hypotonia in infancy with later ataxia; and 3) developmental delay/intellectual disability [29].

MTS is visualized on an axial view by an MRI through midbrain, pons and mesencephalon [2, 30]. MTS is associated with hypoplasia of the cerebellar vermis, a characteristic feature of JS, which is principally accessible prenatally [31].

The hypotonia in infancy of JS patients gives way to later ataxia. It has been shown that developmental delay in the motor system and cognitive development with emotional and behavior problems are typically observed in patients with JS [32]. In connection with hypotonia, abnormal respiratory behavior in infancy, such as tachypnea/apnea, are typically observable [33] and may even be life-threatening, typically under the age of five years [34]. Also typical for patients with JS are oculomotor apraxia, nystragmus, strabismus, and ptosis of eyelids. JS is also characterized by delayed visual development and variable visual acuity [14, 17]. Renal diseases and hepatic fibrosis are associated with JS, as well; renal failure can typically be fatal over five years of age [34]. Other impairments can include oral or tongue hamartomas, skeletal dysmorphies, and occipital encephalocele [6, 35].

### Molecular diagnose of Joubert syndrome

**Figure 1:**
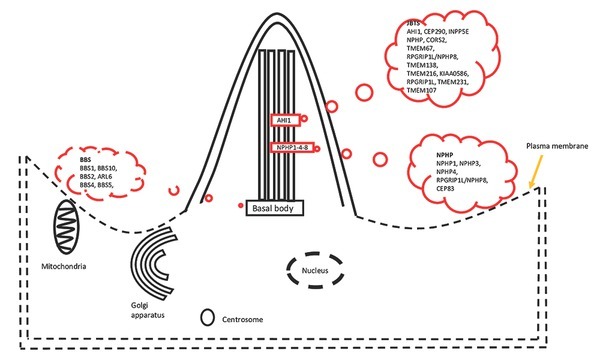
Renal ciliopathies like Joubert syndrome (JS), Bardet-Biedl syndrome (BBS) and nephronophthsis (NPHP) and the known reported genes associated with them are shown in context of the human cell. All of the genes/ their products are connected to basal body and/ or cilia.

JS is inherited with a predominantly autosomal recessive inheritance pattern, with few cases being inherited as an X-linked disorder. However, causative mutations for JS are reported for >35 genes (*INPP5E, TMEM216, AHI1, NPHP1, CEP290 (NPHP6), TMEM67 (MKS3), RPGRIP1L, ARL13B, CC2D2A, OFD1, TTC21B, KIF7, TCTN1, TCTN2, TMEM237, CEP41, TMEM138, C5orf42, TCTN3, ZNF423, TMEM231,CSPP1*, and *PDE6D*), which lead to different phenotypic variations (see Figure 1) [3,53]. Between 50-92% of JS-patients have biallelic pathogenic variants of overlapping genes; in 40-70% of JS-families, 5-6 genes such as *AHI1*, *CCD2D2A*, *CEP290*, *CPLANE1*, *KIAA0586* and *TMEM67* may carry mutations and provide an outbreak of JS [6]. Thus, diagnostics of JS can best be completed by next-generation sequencing, using corresponding gene panels. For different ethnic groups, the panels need to be adapted. For example, in the Ashkenazi population the TMEM216 p.R73L* is the most common mutation, while in the Hutterites community, the most common variant is TMEM237 pR18* [9, 36]. Most typical for Iranian families is *TMEM67 p.Asn242Ser* [37], while in Japanese families, several common variants gene *TMEM67* are found, such as p.Arg110Gly; p.Ser771Pro; p.Arg764*; p.Gly132Ala; p.Ser159Pro; p.Met252Thr; p.Tyr513*; p.Ala145Ser [15] **(Table 1**).

**Table 1:** The most frequent variant gene mutation in patients with Joubert syndrome in Ashkenazi, Hutterites community, Iranian, Japan and Kosovo populations

Gene/Locus	Amino acid change	Population	Reference
*TMEM216*	p.R73L	Ashkenazi population	[9,36]
*TMEM237*	pR18	Hutterites community	[9,36]
*TMEM67*	p.Asn242Ser	Iranian families	[37]
*TMEM67*	p.Arg110Gly;	Japanese	[15]
	p.Ser771Pro;		
	p.Arg764*;		
	p.Gly132Ala;		
	p.Ser159Pro;		
	p.Met252Thr;		
	p.Tyr513*;		
	p.Ala145Ser		
*CEP290*	c.5493delA, p.(A1832fs*19)	Kosovo	[43]

Some genes found to be causative for JS as well as eye, renal and hepatic diseases are presented in **Table 2**.

**Table 2:** Other gene mutation in patients with Joubert syndrome besides those given in Table 1.

Gene/Locus	Position mutated	Amino acid change	Reference
*TMEM216*		p.R73L; p.R73C	[9, 54]
*TMEM237*		pR18	[36]
*TMEM67*		p.Asn242Ser	[37]
*TMEM67*		p.Arg110Gly; p.Ser771Pro; p.Arg764*; p.Gly132Ala; p.Ser159Pro; p.Met252Thr; p.Tyr513*; p.Ala145Ser	[15]
*KIAA0586 (orthologue of TALPID3)*	c.230C>G	p.Ser77*	[44]
*KIAA0586 (orthologue of TALPID3)*	c.230C>G		[44]
*TALPID3*	c.428delG		[45]
*RPGRIP1L*	c.1810G>A	p.Glu604Lys	[46]
*TMEM67/ RPGRIP1L*	c.6012-12T>A		[15]
*TMEM138/ BBS1*	c.6012-12T>A		[15]
*INPP5E*	c.1064C>T	p.T355M	[47]
*TTC21B*	c.2258C>T	p.P753L	[47]
*INPP5E*	c.1565G>C	p.Gly552Ala	[48]
*AHI1*	c.703dupA	p.Arg235LysfsTer12	[49]
*AHI1*	c.2212C>T	p.Arg738Ter	[49]
*AHI1*		p.Thr304AsnfsX6	[50]
*AHI1*	c.832C>T	p.Gln278Ter	[51]
*BBS2*	c.899A>G, c.1814C>G, c.2107C>T		[52]
*INPP5E*	c.1073C>T, c.1669C>T		[52]
*CACNA1F*	c.3582C>G, c.5704-5C>G		[52]

### Genetic counseling

Genetic counseling by a clinical geneticist is imperative to help JS families understand mechanisms of inheritance, repetition risk, reproductive options, and genetic testing. In vitro fertilization with preimplantation testing for known pathogenic variants, heterologous insemination, and/or prenatal testing (including prenatal imaging and invasive diagnostics) are possible options to avoid repetition in a family.

In autosomal recessive inheritance, parents of children diagnosed with JS have a 25% risk for each pregnancy, and unaffected siblings have a 67% risk of being carriers. The carrier frequency of JS gene mutations is approximately 1/500; based on this data, the risk of a carrier to have an affected child is <1/2000. Inheritance of JB usually is autosomal recessive, but rarely it may be X-linked recessive. In this concept, each sib of an affected individual has a 25% chance of being affected, a 50% chance of being an asymptomatic carrier, and a 25% chance of being unaffected and not a carrier [3].

Although little is known about fertility in people who are affected by JS, some affected females have had children later in life; thus, teenagers and adults affected by JS should be counseled and be informed on this rare recessive condition and their up-to-50% risk of having affected offspring in the case of meeting another heterozygote JS-gene carrier (pseudo-dominance) [55]. Oligogenic inheritance has been shown to play a pivotal role in ciliopathies [56,57], however, it has not been reported in JS [58,59].

### The future direction of therapies for Joubert syndrome

There is still a lack of information and knowledge about the role of many cilia proteins. Still, there remains hope for the development of therapies to influence specific ciliary phenotypes. A cure for cerebral malformations that underline the MTS may not be achievable, but interventions to ameliorate the co-occurring morbidities such as retinal, renal and liver diseases could significantly impact the quality of life for JS patients.

One way is to treat the cystic renal diseases of NHPH with elevated levels of cAMP and defects in urinary concentrating ability by the use of this drug in clinical practice [38]. Other possibilities are treatment of NPHP including CDK inhibitors, SHH agonists and mTOR pathway inhibitors such as rapamycin, many of which are still in the early phase of development [21]. Gene therapy is a great promise for treatment of cystic renal diseases associated with JS: ASO (anti sense oligonucleotide) has been used to promote alternative mRNA splicing and exon skipping to save an intronic mutation in gene *CEP290* in patients with renal defects and in a mouse model of JS, when it came to the reduction of cysts in kidney diseases [39]. Similarly, results obtained in treatment of patients with LCA (Leber Congenital Amaurosis) applying gene therapy have demonstrated some success in a cell model and in mice [40]. In conclusion, there are novel strategies emerging to treat these and other manifestation of ciliopathies [41, 42].

## Conclusions

During the last decade, tremendous progress in understanding the genetic causes of JS have been achieved, including the definition of the phenotype spectrum and association with specific genes (see Tables 1 and 2). In the case of recognition of MTS, NGS allows for characterization of the underlying genetic cause in up to 94% of cases; accurate prognosis enables recommendation for surveillance of affected organs and to improve genetic counseling for members of affected families. There is still a lot of work to do, including identification of further JS-causing genes and improved understanding of involved metabolic pathways with the long-term goal to optimize treatment options for affected patients.
